# Community-based molecular and serological surveillance of subclinical malaria in Myanmar

**DOI:** 10.1186/s12916-021-01993-8

**Published:** 2021-05-28

**Authors:** Katherine O’Flaherty, Win Han Oo, Sophie G. Zaloumis, Julia C. Cutts, Kyaw Zayar Aung, Myat Mon Thein, Damien R. Drew, Zahra Razook, Alyssa E. Barry, Naanki Parischa, Nyi Nyi Zaw, Htin Kyaw Thu, Aung Thi, Wai Yan Min Htay, Aung Paing Soe, Julie A. Simpson, James G. Beeson, Paul A. Agius, Freya J. I. Fowkes

**Affiliations:** 1 Burnet Institute for Medical Research and Public Health, Melbourne, Australia; 2 Centre for Epidemiology and Biostatistics, Melbourne School of Population and Global Health, The University of Melbourne, Melbourne, Australia; 3 Burnet Institute Myanmar, Yangon, Myanmar; 4 Department of Medicine, University of Melbourne, Melbourne, Australia; 5 School of Medicine, Deakin University, Geelong, Australia; 6 Department of Public Health, Myanmar Ministry of Health, Nay Pyi Taw, Myanmar; 7 Department of Microbiology and Central Clinical School, Monash University, Melbourne, Australia; 8 Department of Epidemiology and Preventative Medicine, Monash University, Melbourne, Australia; 9 Judith Lumley Centre, La Trobe University, Melbourne, Australia; 10 Department of Infectious Diseases, Monash University, Melbourne, Australia

**Keywords:** Malaria, *Plasmodium*, Surveillance, Epidemiology, Serosurveillance, Immunity

## Abstract

**Background:**

In the Greater Mekong Subregion (GMS), current malaria surveillance strategies rely on a network of village health volunteers (VHVs) reporting the results of rapid diagnostic tests (RDTs), known to miss many asymptomatic infections. Integration of more sensitive diagnostic molecular and serological measures into the VHV network may improve surveillance of residual malaria transmission in hard-to-reach areas in the region and inform targeted interventions and elimination responses. However, data on residual malaria transmission that would be captured by these measures in the VHV-led testing and treatment surveillance network in the GMS is unknown.

**Methods:**

A total of 114 VHVs were trained to collect dried blood spots from villagers undergoing routine RDTs as part of VHV-led active and passive case detection from April 2015 to June 2016. Samples were subjected to molecular testing (quantitative polymerase chain reaction [qPCR]) to determine *Plasmodium falciparum* and *P. vivax* infection and serological testing (against *P. falciparum* and *P. vivax* antigens) to determine exposure to *P. falciparum* and *P. vivax*.

**Results:**

Over 15 months, 114 VHVs performed 32,194 RDTs and collected samples for molecular (*n* = 13,157) and serological (*n* = 14,128) testing. The prevalence of molecular-detectable *P. falciparum* and *P. vivax* infection was 3.2% compared to the 0.16% prevalence of *Plasmodium* spp. by RDT, highlighting the large burden of infections undetected by standard surveillance. Peaks in anti-*P. falciparum*, but not *P. vivax*, merozoite IgG seroprevalence coincided with seasonal *P. falciparum* transmission peaks, even in those with no molecularly detectable parasites. At the individual level, antibody seropositivity was associated with reduced odds of contemporaneous *P. falciparum* (OR for *Pf*CSP 0.51 [95%CI 0.35, 0.76], *p* = 0.001, *Pf*AMA1 0.70 [95%CI 0.52, 0.93], *p* = 0.01, and *Pf*MSP2 0.81 [95%CI 0.61, 1.08], *p* = 0.15), but not *P. vivax* infection (OR *Pv*AMA1 1.02 [95%CI 0.73, 1.43], *p* = 0.89) indicating a potential role of immunity in protection against molecular-detectable *P. falciparum* parasitaemia.

**Conclusions:**

We demonstrated that integration and implementation of sample collection for molecular and serological surveillance into networks of VHV servicing hard-to-reach populations in the GMS is feasible, can capture significant levels of ongoing undetected seasonal malaria transmission and has the potential to supplement current routine RDT testing. Improving malaria surveillance by advancing the integration of molecular and serological techniques, through centralised testing approaches or novel point-of-contact tests, will advance progress, and tracking, towards malaria elimination goals in the GMS.

**Supplementary Information:**

The online version contains supplementary material available at 10.1186/s12916-021-01993-8.

## Background

The emergence of artemisinin-resistant *Plasmodium falciparum* within the Greater Mekong Subregion (GMS) has led to the region setting elimination targets for all human malaria by 2030 [1]. In working towards this goal, the incidence of malaria cases and deaths in the GMS fell substantially by 75% and 93%, respectively, between 2012 and 2017 [2]. Monitoring and surveillance are critical to the elimination of malaria ensuring that progress towards malaria elimination targets can be accurately tracked and ultimately accelerated. However, as the region transitions towards malaria elimination, surveillance becomes increasingly difficult because malaria becomes concentrated in discrete geographical foci, such as border and hard-to-reach areas, and in high-risk populations such as migrant workers and residents of highly forested areas [3–5]. To capture these infections, in many remote areas, surveillance and malaria control strategies are dependent on passive case detection (PCD) and active case detection (ACD) provided by a village health volunteer (VHV) network who administer malaria testing by rapid diagnostic test (RDT) as well as treatment. However, subclinical *Plasmodium* spp. infections often go undetected because asymptomatic individuals are less likely to seek testing and treatment and, importantly, are generally below the detection limit of conventional RDT diagnostics used in the field. Undetected, and therefore untreated, *Plasmodium* spp. infections may be an important source of residual malaria transmission [6–10], and failure to detect and eliminate all infections may hinder malaria elimination targets*.*

Integration of more sensitive diagnostic measures into the VHV network may improve surveillance of residual malaria transmission in hard-to-reach areas in the region. Molecular and serological assays can determine residual malaria transmission not detected by routine diagnostics in the field such as RDT and microscopy. Microscopy is estimated to miss approximately 50% of infections when compared to molecular methods such as polymerase chain reaction (PCR), and the proportion of missed infections may be greater than 80% in areas of low transmission (defined at PCR prevalence <10%) [11]. However, the application of sensitive molecular methods such as PCR to detect malaria is mostly utilised as a research tool in many malaria-endemic settings and is yet to be approved and incorporated into routine surveillance in the GMS. Similarly, measuring antibodies specific for malarial antigens is not approved for routine use and may also be a useful surveillance tool to monitoring ongoing malaria transmission in regions approaching malaria elimination as it has the potential to measure both current and recent malaria exposure [12]. Until point-of-contact molecular and serological surveillance tools for malaria become more widely available and approved for use in national malaria control programmes, centralised use of these approaches will be necessary. However, sample collection for surveillance activities may be implemented at the village level by VHV. To date, few studies have been performed in Southeast Asia to investigate the utility of molecular and serological surveillance, and none has been incorporated into the VHV network. Serial cross-sectional research surveys have demonstrated higher blood-stage antimalarial IgG levels and seropositivity amongst those with PCR-detectable subclinical *P. falciparum* and *P. vivax* infection compared to uninfected individuals [13–16], and geospatial analysis has shown that antimalarial antibodies are predictive of ongoing malaria transmission [17]. While these studies suggest the use of serological surveillance may be appropriate for the detection of residual malaria transmission, the feasibility of integrating the approach and the data on residual malaria transmission that would be captured into the PCD/ACD VHV-led testing and treatment surveillance network in the GMS is unknown. To address this knowledge gap, we integrated the collection of participant samples for molecular and serological surveillance into VHV-delivered community-based malaria programmes in Southeast Myanmar to understand the surveillance data that can be captured at this level to inform surveillance of malaria and targeted interventions in elimination settings in the GMS.

## Methods

### Study design and sample collection

Details of the study design and sample collection have previously been reported [18, 19] and are detailed in the Supplementary Methodology (Additional File 1). Briefly, from April 2015 to June 2016, residents of 114 villages across three south-eastern states/regions (Kayin, Kayah and Bago East) in Myanmar were invited to take part in an open stepped-wedge cluster-randomised control trial designed to estimate the effectiveness of topical insect repellent distributed by VHV on *Plasmodium* spp. infection (ACTRN12616001434482). This trial was an implementation trial, and the only changes to the malaria programme implementation were the distribution of repellent at a designated month and the addition of dried blood spot sample collection. Like many areas of the GMS, the incidence of malaria cases in Myanmar has reduced substantially in recent years (by 82% between 2012 and 2017 and by 23% between 2016 and 2017 alone) [20]. Additionally, large proportions of infections in Myanmar are subclinical and not detected using routine RDT [4, 21–23]. Consistent with routine services provision, VHVs performed RDTs (SD bioline *Pf/Pv* combo RDT), treated malaria cases if RDT was positive and collected basic demographic information for the duration of the trial. VHVs aimed to perform a minimum of 20 tests per month, collected by either PCD or ACD, where PCD refers to villagers presenting to the VHV for testing and ACD refers to VHV seeking villagers for testing (e.g. during health education sessions or household visits). VHV also received 1-day training to perform additional finger prick sample collection. In consenting participants, VHVs collected two drops of blood on Whatman 3-mm filter paper for molecular *Plasmodium* spp. detection by quantitative PCR (qPCR) and IgG analysis. Filter papers were air-dried, placed in air-tight plastic bags and then stored in specimen collection boxes. At the end of each month, specimen boxes were collected from VHVs by local dioceses malaria officers and stored in refrigerated conditions. Every 2–3 months, samples were transported to Yangon, where they were stored in the refrigerator before shipment to Melbourne, Australia. All participants or parents/guardians provided informed consent, and ethical approval was obtained from the Ethics Review Committee on Medical Research involving Human Subjects, Myanmar Department of Medical Research (21/Ethics/2015), and the Alfred Hospital, Melbourne, Australia (95/15).

### Outcome measures

#### *P. falciparum* and *P. vivax* infection

*P. falciparum* or *P. vivax* (or both) infection was determined by SD bioline *Pf/Pv* combo RDT according to the manufacturer’s instructions and by quantitative PCR (qPCR) as previously described [19] and detailed in Additional File 1 and 2. A positive *P. falciparum* result included any qPCR-detectable infection where *P. falciparum* was detected, including mixed infections, and likewise for *P. vivax.*

#### *P. falciparum* and *P. vivax* antigen-specific IgG

Total IgG in response to *P. falciparum* apical membrane antigen 1 (*Pf*AMA1 [3D7]), merozoite surface protein 2 (*Pf*MSP2 [3D7]) and circumsporozoite (*Pf*CSP [3D7]) and *P. vivax* AMA1 (*Pv*AMA1 [Palo Alto]) was measured by enzyme-linked immunosorbent assay (ELISA) using a robotic liquid handling system (JANUS automated work station, Perkin Elmer), as previously described [24]. Detailed protein expression and ELISA methodology are provided in the Supplementary Methodology (Additional File 1).

### Statistical analysis

Logistic mixed effects modelling was used to estimate the change in IgG seropositivity (seropositive/seronegative) with time (months) and linear mixed effects modelling was used to estimate the change in Log_2_ IgG level (OD 450nm) with time on individual-level observations. The association between *P. falciparum* or *P. vivax* infection detected by qPCR and the following factors were examined using logistic mixed effects modelling: (i) participant IgG level/seropositivity at the time of detection of malaria infection (contemporaneous IgG level/seropositivity) as separate exposures and (ii) participant IgG level/seropositivity at the preceding presentation (lagged IgG level/seropositivity) as independent exposures. Due to a smaller number of *P. vivax* events in the sample with repeated measurements, only contemporaneous IgG level/seropositivity exposures were investigated. Likelihood ratio tests were used to assess whether (i) time as a discrete factor was associated with odds of IgG seropositivity and Log_2_ IgG levels and (ii) an interaction existed between *P. falciparum* or *P. vivax* infection detected by qPCR and time. All mixed effects models included a random effect (intercept) to account for participant repeated measurement, and covariates for time (discrete month), age (years), distribution of topical insect repellent (time-varying monotonic variable as villages transitioned from control to intervention), residential status (village resident/migrant/forest-dwelling resident), sex (male/female) and the operating diocesan areas (Hpa-An and Taungoo Areas in Kayin State, Loikaw Area in Kayah State, Yangon Area in Bago East Region). Models estimating lagged IgG level/seropositivity excluded fixed effects for discrete time (due to model overfit) but included a fixed effect for the time between visits (number of days) for each individual. All data were analysed using Stata14 (StataCorp, Stata Statistical Software: College Station, TX).

## Results

### Characteristics of the study population

One hundred and fourteen VHVs (48% male, median age 26 years (25th and 75th percentiles 21–33) in 102/114 VHV respondents) were trained to undertake malaria diagnosis by RDT and collection of dried blood spot samples in their villages. From April 2015 until June 2016, trained VHVs performed 32,194 RDTs in 114 villages of Southeast Myanmar, as previously described [19]. In a sub-population of consenting participants, VHVs collected 14,938 dried blood spot samples, 810 of which were missing key demographic data (*n* = 692) or were compromised due to improper storage (*n* = 118) and were excluded from all further molecular and serological analyses. The final sub-sample included 14,128 samples from 10,857 individuals (Table 1). In this population, the median age of participants was 20 years (25th and 75th percentiles 10–35, min and max <1–91), and 49.8% were male (5402/10,857). Of all participant samples, 13.1 % were collected from migrants (1423/10,857), 39.9 % were collected from village residents (4329/10,857) and 47 % were collected from forest dwellers (5105/10,857) (Table 1). The sub-population that consented to sample collection was broadly reflective of the larger cohort with RDT diagnosis only (Table 1). The majority of participants (81.3%, *n* = 8825/10,857) provided only one sample throughout the study period (Table S3). There were 2032 participants that contributed multiple samples (total samples = 5303) with a median of two samples per person (25th and 75th percentiles 2–3, min and max 2–10 samples per person) with a median time between sampling of 118 days (25th and 75th percentile 50–168 days, min and max 4–442 days).

**Table 1:** Characteristics of VHV performed RDTs and DBS

	Total population	Population with RDT only	Population with RDT and DBS
Number of participants	–^a^	–^a^	10,857
Number of RDTs	32,194	18,066	14,128
Age (years), median (25th, 75th percentile; min, max)	18 (9, 33; 0.1, 100)	16 (8–31, 0.1–100)	20 (10–35, 0.1–91)
Sex, % male (*n*/*N*)	50.06 (16,116/32,194)	50.49 (9122/18,066)	49.76 (5402/10,857)
Residential status^b^, % (*n*/*N*)			
Migrant	13.71 (4415/32,191)	15.74 (2844/18,063)	13.11 (1423/10,857)
Village resident	45.92 (14,783/32,191)	48.85 (8826/18,063)	39.87 (4329/10,857)
Forest dweller	40.36 (12,993/32,191)	35.39 (6393/18,063)	47.02 (5105/10,857)
RDT, % (*n*/*N*)			
Negative	99.84 (32,144/32,194)	99.83 (18,036/18,066)	99.86 (14,108/14,128)
*Pf*+	0.04 (13/32,194)	0.03 (6/18,066)	0.05 (7/14,128)
*Pv*+	0.11 (34/32,194)	0.12 (22/18,066)	0.08 (12/14,128)
Mixed	0.01 (3/32,194)	0.01 (2/18,066)	0.01 (1/14,128)
qPCR^c^, % (*n*/*N*)			
Negative	N/A	N/A	96.82 (12,738/13,157)
*Pf*+	N/A	N/A	1.57 (207/13,157)
*Pv*+	N/A	N/A	0.93 (123/13,157)
Mixed	N/A	N/A	0.68 (89/13,157)

*DBS* dried blood spot, *IQR* interquartile range, *Pf Plasmodium falciparum*, *Pv Plasmodium vivax*, *qPCR* quantitative polymerase chain reaction, *R* range, *RDT* rapid diagnostic test, *VHV* village health volunteer

^a^Individual participant identifiers were only given to consenting participants upon DBS sampling

^b^Residency data missing for 3 participants

^c^qPCR detection only performed in participants consenting to DBS collection and N/A for 971 due to insufficient sample

### Testing rates and detection of *P. falciparum* and *P. vivax* infection by RDT and qPCR over time

*P. falciparum* and *P. vivax* infection status was determined in all participants by RDT (*n* = 32,194), and 14,128 samples were collected for molecular and serological analysis. From this sub-sample, 13,157 samples were suitable for molecular diagnosis by qPCR, and 971 were excluded due to insufficient sample (Table 1). As reported previously [19], the prevalence of *Plasmodium* spp. infections detected by RDT was 0.16% (50/32,194; 13 *P. falciparum* mono-infection [0.04%], 34 *P. vivax* mono-infection [0.11%] and 3 mixed species infections [0.01%], 29 symptomatic), and in the sub-sample of participants consenting to additional sampling, RDT-positive *Plasmodium* spp. infection was similarly low at 0.14% (20/14,128; 7 *P. falciparum* mono-infection [0.05%], 12 *P. vivax* mono-infection [0.08%] and 1 mixed species infections [0.01%]) (Table 1). In this sub-sample of participants, 13,157 underwent diagnosis by qPCR, and the prevalence of *P. falciparum* and *P. vivax* infection was 3.2% (419/13,157; 207 *P. falciparum* mono-infection [1.57%], 123 *P. vivax* mono-infection [0.93%] and 89 mixed species infections [0.68%]), approximately 22-fold higher than by RDT (Table 1).

**Fig. 1:**
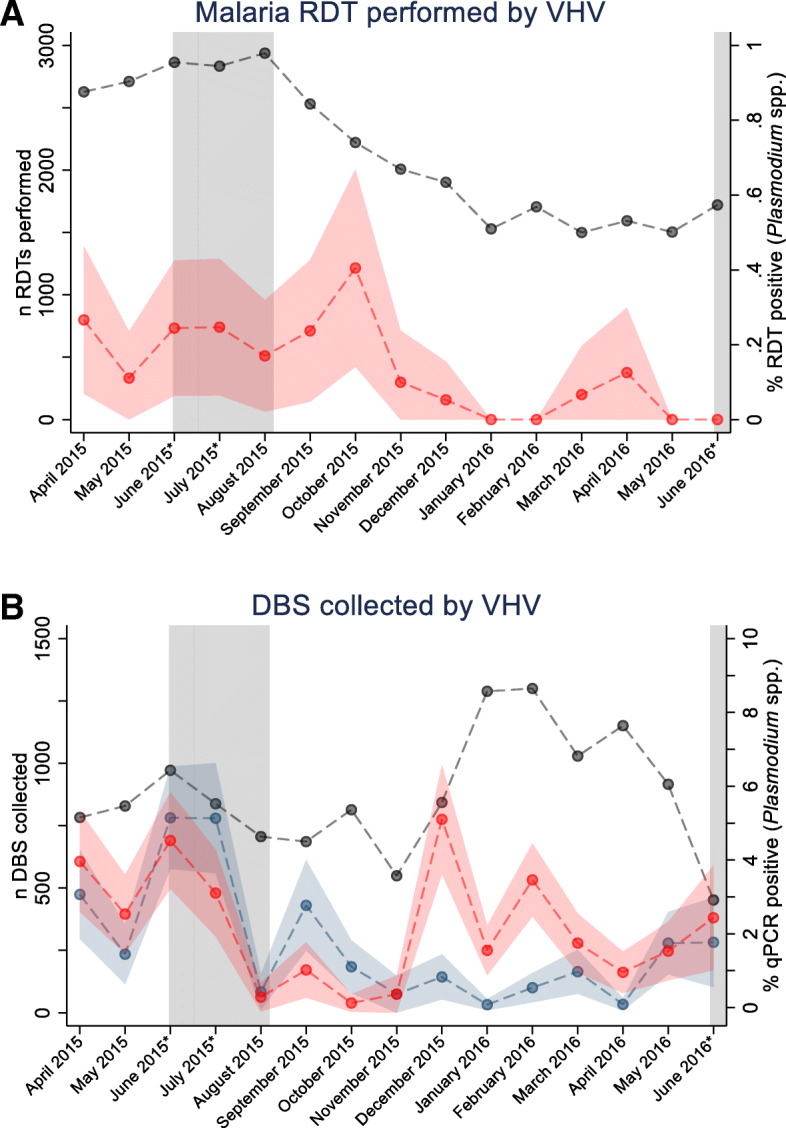
Number of RDTs collected by VHV (grey) and prevalence of *Plasmodium* spp. infection by RDT (95% CI [red]) (**a**) and number of DBS collected by VHV (grey) and prevalence of *Plasmodium* spp. infection (95% CI) by qPCR (*P. falciparum* [red] and *P. vivax* [blue]) (**b**) by month over the study period. The high transmission season is shown in grey and months marked with an asterisk. Lines connecting data points are intended only to highlight patterns and not to suggest a continuum between data points

The average testing rate was 2146 (standard deviation [SD] 554) RDTs per month for the entire VHV network over the course of the study (20 [SD 14] RDTs per village/VHV per month); however, this varied by month, with 2628 RDTs/month in the first month of the study decreasing to 1721 RDTs/month in the last month of the study (Fig. 1a). The average number of dried blood spots collected was 942 (SD 201) samples per month (12 [SD 8] samples per village/VHV per month), with more samples collected per month occurring in the latter part of the study (Fig. 1b). Both qPCR-detectable *P. falciparum* and *P. vivax* prevalence varied over time (discrete month, likelihood ratio [LR] both *p*<0.001) (Fig. 1). *P. falciparum* and *P. vivax* infection was observed to peak in June and July corresponding to the high transmission season in Myanmar (9.67% and 8.23% in June and July, respectively) with a second, smaller peak observed in December for *P. falciparum* (5.93%, Fig. 1b).

### Seroprevalence and levels of antimalarial IgG over time

**Fig. 2:**
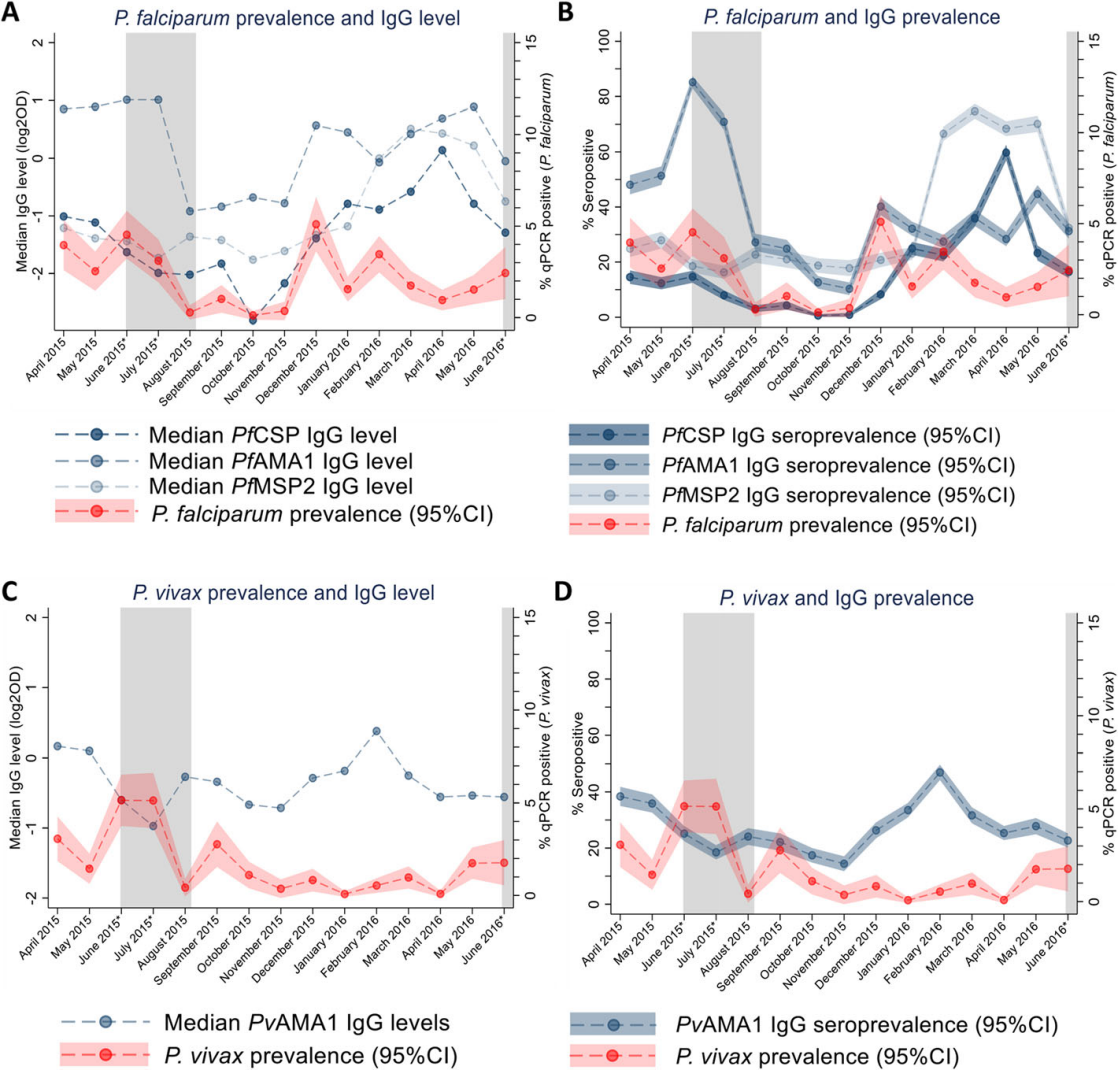
Prevalence of *P. falciparum* and *P. vivax* infection and levels and seroprevalence of anti-*P. falciparum* and *P. vivax* IgG by month over the study period. Prevalence of *P. falciparum* (**a**, **b**) and *P. vivax* (**c**, **d**) infection and median IgG level (**a**, **c**) and seroprevalence (95%CI) (**b**, **d**) IgG by month over the study period. The high transmission season is shown in grey and months marked with an asterisk

To determine the change in the acquired IgG levels and seroprevalence over the study period, IgG responses to *P. falciparum* merozoite and sporozoite antigens *Pf*AMA1, *Pf*MSP2 and *Pf*CSP, and *P. vivax* merozoite antigen *Pv*AMA1 were determined. Because elution of antibody required less sample than DNA extraction, IgG levels and seroprevalence were determined in all samples collected that had not been compromised and had matched data (*n *= 14,128). The overall seroprevalences for *Pf*AMA1, *Pf*MSP2, *Pf*CSP and *Pv*AMA1 were 38.6 (95% CI 37.8–39.4), 37.3 (95% CI 36.5–38.1), 18.7 (95% CI 18–19.3) and 28.4% (95% CI 27.6–29.1), respectively. Both anti-*P. falciparum* and *P. vivax* IgG level and seroprevalence were associated with time (discrete month, LR all *p* <0.001, Table S4 – S11). Like patterns of qPCR-detectable *P. falciparum* infection, the median levels and overall seroprevalence of anti-*Pf*AMA1 IgG in all observations peaked in June and July of 2015 coinciding with the high transmission season, with an additional peak observed in December of 2015 coinciding with a peak in qPCR detectable *P. falciparum* cases. Peaks in *Pf*MSP2 and *Pf*CSP IgG were only observed to coincide with the second peak in *P. falciparum* infections in December of 2015 (Fig. 2a, b). Interestingly, these seasonal trends in *P. falciparum* specific IgG seroprevalence were also reflected in a broader qPCR-negative population (Additional File 2 Figure S4). The median levels and seroprevalence of anti-*Pv*AMA1 IgG fluctuated and increased slightly throughout the study period; however, these changes were largely unreflective of changes in qPCR detected *P. vivax* prevalence (Fig. 2c, d).

### Association between IgG and *P. falciparum* and *P. vivax* infection

To determine the association between IgG seropositivity on *P. falciparum* and *P. vivax* qPCR-detectable infection at the individual level, we performed logistic mixed effects modelling, including all data and fixed effects for age, sex, region, residential status, time and repellent distribution, and random effects for repeated participant sampling. Seropositivity for anti-*Pf*AMA1, *Pf*MSP2 and *Pf*CSP IgG was associated with a reduction in the odds of contemporaneous qPCR-detectable *P. falciparum* infection by between 19 and 49%;, however, a wide confidence interval was observed in the *Pf*MSP2 estimate (anti-*Pf*AMA1 OR 0.70 (95%CI 0.52, 0.93), *p* = 0.01, anti-*Pf*MSP2 OR 0.81 (95%CI 0.61, 1.08), *p* = 0.15, anti-*Pf*CSP OR 0.51 (95%CI 0.35, 0.76), *p* = 0.001; Table 2). Similarly, a twofold increase in the levels of anti-*Pf*MSP2 and *Pf*CSP were associated with a 12 and 10% reduction, respectively, in the odds of *P. falciparum* infection (*Pf*MSP2 OR 0.88 (95%CI 0.80, 0.96), *p* = 0.005, anti-*Pf*CSP OR 0.90 (95%CI 0.83, 0.98), *p* = 0.02, Table 2). Increased anti-*Pf*AMA1 IgG level was not associated with the odds of qPCR-detectable *P. falciparum* infection (OR 0.94 (95%CI 0.84, 1.06), *p* = 0.34, Table 2). When lagged, IgG level/seroprevalence was examined as the exposure (i.e. using the preceding IgG measurement), in participants with repeated measurements after including a fixed effect for the time between sampling (days) anti-*P. falciparum* IgG level/seropositivity was not associated with qPCR-detectable *P. falciparum* (Table 2). Anti-*Pv*AMA1 IgG level and seropositivity were not associated with contemporaneous qPCR-detectable *P. vivax* infection (OR 1.02 (95%CI 0.73, 1.43), *p* = 0.89 and OR 0.98 (95%CI 0.86, 1.12), *p* = 0.75, Table 2, respectively). Due to a smaller number of *P. vivax*-positive participants with repeated measurements (*n*=68), lagged effects of IgG were not estimated.

**Table 2:** Association between qPCR-detectable *P. falciparum* or *P. vivax* infection and antibody response

	Contemporaneous infection			Infection at next presentation		
	aOR^a^	(95%CI)	*p* value	aOR^b^	(95%CI)	*p* value
*Pf*AMA1 IgG						
Seropositive	0.70	(0.52, 0.93)	0.01	0.98	(0.53, 1.80)	0.95
Level (log_2_OD 450nm)	0.94	(0.84, 1.06)	0.34	0.91	(0.73, 1.10)	0.33
*Pf*MSP2 IgG						
Seropositive	0.81	(0.61, 1.08)	0.15	1.27	(0.70, 2.29)	0.43
Level (log_2_OD 450nm)	0.88	(0.80, 0.96)	0.005	0.92	(0.77, 1.11)	0.40
*Pf*CSP IgG						
Seropositive	0.51	(0.35, 0.76)	0.001	0.29	(0.07, 1.21)	0.09
Level (log_2_OD 450nm)	0.90	(0.83, 0.98)	0.02	1.02	(0.85, 1.24)	0.81
*Pv*AMA1 IgG^c^						
Seropositive	1.02	(0.73, 1.43)	0.89	–		
Level (log_2_OD 450nm)	0.98	(0.86, 1.12)	0.75	–		

Adjusted for ^a^age (years), sex, region, residential status, time (discrete month) and repellent distribution or ^b^age (years), sex, region, residential status, time since the last measurement (days) and repellent distribution; ^c^only contemporaneous IgG level/seropositivity exposures were estimated for *P. vivax* infections due to a small number of events for individuals with measurements performed on at least two occasions. Estimates for antigen- specific IgG seropositivity or level are derived from separate equations, (Additional File 2 Tables S3-S10)

*aOR* adjusted odds ratio, *95%CI* 95% confidence interval

## Discussion

VHVs are the cornerstone of malaria control and surveillance activities in many malaria-endemic settings. By training VHV delivering routine malaria services to integrate sample collection for molecular and serological surveillance in their village, we demonstrated that surveillance using highly sensitive molecular methods in samples collected by VHV is feasible and can detect a significant subclinical reservoir of infection undetected by standard surveillance using RDTs. Furthermore, serological data reflected monthly trends in *P. falciparum* and *P. vivax* infection, even in those with no detectable parasites, indicating that this surveillance approach can capture significant levels of ongoing undetected seasonal malaria transmission. Integration of molecular and serological surveillance into the extensive VHV network may be an effective tool for monitoring residual malaria transmission in hard-to-reach pre-elimination settings by supplementing an established VHV surveillance network currently dependant on RDTs. Further, the addition of this kind of supplemented surveillance could support centralised malaria control strategies such as resource allocation until more suitable field-deployable tests are available to provide VHV with real-time treatment decision-making tools. Improving malaria surveillance will advance progress, and tracking, towards malaria elimination goals in the GMS.

RDT diagnosis and reporting of malaria cases by VHVs is the key indicator for progressing towards malaria elimination goals in the GMS. Like in much of the region, a large proportion of *Plasmodium* spp. infections found in participating villages were missed by RDT diagnosis with ~22-fold greater prevalence of qPCR-detectable infections over the entire study period. There was significant variation in qPCR-detectable infection over time with peaks of *Plasmodium* spp. infection in the rainy season, where malaria transmission increases, which were not detected by RDT, nor subsequently treated. There is increasing evidence that these untreated molecular-detected infections have the potential to contribute to ongoing malaria transmission, and despite not being transmitted as effectively to mosquitoes as microscopically detected infections, the greater prevalence of subclinical compared to clinical infections in low-transmission settings is cause for concern [25]. Detection of these infections is becoming increasingly difficult because in the elimination phase, malaria is concentrated in hard-to-reach areas and high-risk populations. Therefore, additional surveillance tools deployed at the VHV level may help target interventions aimed at reducing the subclinical burden of malaria and advance the malaria elimination agenda in this region.

Serosurveillance does not provide a diagnosis of a current *Plasmodium* spp. infection. Rather, at a population level, antimalarial antibodies may indicate recent *Plasmodium* spp. transmission and exposure and therefore represent a useful tool for malaria microstratification to inform resource allocation and targeted implementation of malaria control and elimination interventions by NMCPs if integrated into national surveillance. The observed peaks in anti-*P. falciparum* merozoite IgG seroprevalence coinciding approximately with peaks of PCR-detectable *P. falciparum* infection, even in parasite negative individuals, indicate that malarial serology captured ongoing malaria transmission in the population not detected by routine RDTs. However, the magnitude of antibody seroprevalence was significantly greater than the prevalence of PCR detectable infections. Caution must be exercised in estimating the prevalence of circulating infections based on serology, which may be overestimated particularly in regions where the malaria burden is transitioning from malaria control to elimination phases, as antibodies can persist for extended periods [26, 27]. This study was conducted amid Myanmar’s transition from malaria control to elimination and following large-scale reductions in *Plasmodium* spp. transmission in the study region [28]. The higher seroprevalence compared to PCR prevalence of *P. falciparum* and *P. vivax* infections may reflect cumulative incidence in the region over this time period [28, 29]. Between 2005 and 2014, immediately prior to the commencement of this study in 2015, the prevalence of malaria detected by RDT declined in Kayah, Kayin and Bago states by more than 74% (from 3.2 to 1.7%, 0.86 to 0.52%, and 0.44 to 0.07%, respectively) [29]. The historical prevalence in the 10 years prior to this study are considerably higher than the 0.16% RDT positivity reported here and may result in the cumulative incidence of antimalarial antibody responses observed because the mean antimalarial antibody half-lives of the antigens included in this study (selected because their immunogenicity and half-lives are relatively well-characterised in the GMS) may range between 6 months and 7 years IgG [27, 30, 31]. As such, serosurveillance may be a more informative tool as antibodies wane in the broader population and is limited to only exposed populations in elimination phases or alternatively different sets of antigens could be defined which can quantitatively capture very recent molecular infection events [14]. This study was not designed to identify serological biomarkers of recent exposure to molecularly detectable infections as this would require detailed longitudinal follow-up and accurate participant exposure history. Several recent studies have demonstrated the utility in combining novel combinations of antigens to predict recent *Plasmodium* spp. infection exposure detected by microscopy, clinical incidence and relapse with *P. vivax*, across diverse malaria-endemic populations [14, 32–34]. However, the efforts to identify reliable, quantitative antibody signatures of recent exposure to malaria parasites are ongoing [35, 36], and there is currently a lack of consensus on antigen-specific antibody responses which discriminate against recent and historical exposures, in particular, of molecularly detectable infections. We investigated *P. falciparum* antigens based on the 3D7 allelic variant. There is limited data on antigen allelic diversity in Myanmar, but previous studies have shown that the majority of circulating MSP alleles are of the 3D7 type [37] and that variation in IgG response to the different allelic variants of *Pf*MSP2 across multiple sites in Myanmar and the Greater Mekong Subregion is minimal [38]. Furthermore, antibody responses specific for epitopes within conserved domains of merozoite antigens have been shown to illicit strain transcending antibody responses [39], so inclusion of additional antigenic variants may not have impacted our study conclusions. However, further refinement of choice and number of antigens and allelic types across different areas of Myanmar and the GMS is warranted to inform the utility of serological findings in the region. Regardless of ultimate antigen selection or malaria burden phase, any new tools need to be validated with respect to the health system platform, such as the VHV-led delivery of malaria services, which will ultimately be integrated into.

In individual-level analyses, we observed a reduction in the odds of concurrent qPCR detectable *P. falciparum* infection in participants seropositive for *P. falciparum*-specific IgG. Recent longitudinal data from the GMS has shown that within infected individuals, parasite density oscillates frequently and that spontaneous clearance of PCR-detectable *P. falciparum* infection (i.e. in the absence of treatment) occurs regularly with the median time to resolution of infection of around 2 months for *P. falciparum* and 6 months for *P. vivax* [40]. We hypothesise that boosting of antibodies upon exposure to parasites may then contribute to clearance of subclinical parasitaemia which may explain the observed reduction in the odds of a contemporaneous qPCR-detectable *P. falciparum* infection in seropositive individuals when sampled in the VHV network. This study provides evidence of potential protective immunity against molecular-detectable infections, which to date has only been established to reduce clinical malaria and high parasitaemia in numerous high-transmission populations [41]. We were unable to show protection against prospective infection; however, there were fewer repeated measures (returning participants were less than 20% of all samples) with large variation in the time between tests. Further studies investigating the response to and role of anti-malarial antibodies in the persistence of molecular-detectable subclinical infections would be valuable to inform the utility and sampling framework of serological surveillance in areas where the majority of the malaria burden remains undetected by conventional surveillance.

The aim of this study was to investigate malaria incidence and serological data within an existing national malaria surveillance strategy, in this case, the VHV network. In the national VHV testing strategy, some individuals will be sampled multiple times and others only once, that is, malaria incidence is representative of the population that is sampled by VHV which may not be truly representative of the general population per se. Repeated sampling of a subset of individuals within the same population risks overestimating malaria incidence when the same individuals present multiple times with a single infection. In this study, which accounted for repeated measures in statistical analyses, only one participant contributing multiple samples was qPCR positive more than once, in samples collected 10 months apart. Given the estimated clearance time of sub-clinical *P. falciparum* infection in the region is estimated to be under 3 months [40], these likely represent two separate infection events. Therefore, in the present study, multiple samples from individual participants would not have biassed estimates of malaria incidence in samples collected by VHV. Nonetheless, accurate estimates of malaria incidence due to repeated sampling of a subset of individuals should be considered in the broader national malaria surveillance strategy, given that the sampling strategy is the same as current RDT testing implemented nationally at the VHV level.

Sampling at the VHV level may facilitate increased surveillance in rural and hard-to-reach areas. In this study, VHVs aimed to collect a minimum of 20 RDTs each month in accordance with programme implementation through PCD/ACD; however, data could not be stratified based on the VHV case detection method as this data was not collected. As such, we are unable to determine whether PCD alone would be sufficient to accurately capture the subclinical malaria burden or changes in serological markers reported here. The total number of RDTs declined across the study period, and this is most likely due to the declining motivation of the VHV to undertake RDTs which has been described in the region in areas approaching elimination where positive RDT results are infrequent [42]. This was not reflected in sample collection, the total number of which fluctuated across the study. Participation in sample collection, however, occurred in less than half of participants receiving testing by RDT which, from anecdotal reports, may have been due to reluctance to commit further time to complete the additional informed consent and testing procedures (in Myanmar, dried blood spot sampling is not part of routine VHV services and considered research; therefore, additional consent is required). However, participants contributing samples were broadly similar to those contributing RDTs. Until dried blood spot sampling is considered part of routine sample collection, routinely collected RDTs could be utilised as an alternative source of sample for molecular and serological surveillance of malaria to overcome participation issues [43–45], but their utility for molecular assays, given low parasite densities, needs to be investigated in the Myanmar context. Importantly, capacity building for local laboratories needs to be prioritised as well as understanding how this data will operationally be integrated into the national surveillance electronic system in order to inform programmatic decisions.

## Conclusions

Current malaria surveillance strategies in the GMS rely on VHV reporting the results of insensitive RDTs that do not detect low-density infections. These undetected, untreated infections may contribute to ongoing malaria transmission and have the potential to hinder progress towards malaria elimination. We have shown that sample collection for molecular and serological surveillance can be integrated and implemented in community-delivered VHV health programmes in hard-to-reach populations and has the potential to capture ongoing malaria transmission at the individual and population levels. While molecular and serological testing in this study was undertaken after fieldwork was completed, away from the villages, data captured by sampling at the VHV level has the potential to inform programmatic decisions such as resource deployment by national malaria control programmes. With the increasing development and availability of easy-to-use, portable point-of-contact molecular and serological tests, there is future potential for VHV to undertake molecular and serological malaria surveillance and report detected cases in near real time. This will not only advance current routine VHV-led malaria surveillance, particularly in hard-to-reach areas, but will also reinforce ownership of malaria surveillance and the malaria elimination agenda to the community who will ultimately play a key role in reaching malaria elimination goals in the GMS.

## Supplementary Information

**Additional file 1.** Supplementary methodology.

**Additional file 2.** Supplementary tables and figures.

## Data Availability

Data cannot be made publicly available because it would breach compliance with the ethical framework of the Ethics Review Committee on Medical Research Involving Human Subjects, Department of Medical Research, Myanmar Ministry of Health and Sports. De-identified individual participant data will be available after publication from the data custodian(s) to applicants who provide a sound proposal to the Ethics Review Committee on Medical Research Involving Human Subjects, Department of Medical Research, Myanmar Ministry of Health and Sports (No. 5 Ziwaka Road, Dagon PO Yangon, Myanmar; (+95) 01 375447 extension 118; moc.liamg@5102rmdcre) contingent of their approval.

## Abbreviations

ACD: Active case detection
AMA1: Apical membrane antigen 1
CSP: Circumsporozoite protein
ELISA: Enzyme-linked immunosorbent assay
GMS: Greater Mekong Subregion
Ig: Immunoglobulin
LR: Likelihood ratio
MSP2: Merozoite surface protein 2
OR: Odds ratio
PCD: Passive case detection
PCR: Polymerase chain reaction
*Pf*: *Plasmodium falciparum*
*Pv*: *P. vivax*
qPCR: Quantitative PCR
RDT: Rapid diagnostic test
SD: Standard deviation
VHV: Village health volunteer
