# Supplementary material for: Community-based molecular and serological surveillance of subclinical malaria in Myanmar
Source: BMC Med. 2021 May 28;19:121. doi: 10.1186/s12916-021-01993-8 (PMC8161608; doi:10.1186/s12916-021-01993-8)
Supplement: Supplementary file 1 — Additional file 1. Supplementary methodology. [file 12916_2021_1993_MOESM1_ESM.docx]

**Supplementary Methodology**

**Study design and sample collection**

Participant samples were acquired from a stepped wedge, cluster randomised control trial exploring the effect of topical insect repellent (N,N-diethyl-benzamide-12% w/w) distributed by village health volunteers (VHVs) on *Plasmodium* spp. infection in high risk populations in Southeast Myanmar, first described by Win Han Oo *et al.* in 2018 (1). Based on services data provided by the Myanmar National Malaria Control Program, 116 villages surrounding eight townships were selected in the South-eastern states Kayin, Kayah, and Bago (Figure S1). Included sites represented isolated villages and hard-to reach territories that lacked malaria services. The study commenced in April of 2015, and following a baseline period of one month, repellent was distributed to high risk populations each month according to randomisation of village blocks (Figure S2). For the purposes of this study, high risk populations were defined as either forest dwelling or migrant people, where a forest dweller referred to any person/s regularly working and/or staying overnight in forested regions, and a migrant referred to any person/s who takes up residence or remains in another place for an extended period of time, including seasonal migrants. The study ceased in June of 2016. The primary endpoint of the study was the incidence of *P. falciparum* and *P. vivax* infection diagnosed by RDT (SD bioline p.f/p.v combo) detected through active and passive detection, assessed at the village level. RDTs were performed monthly within each village by VHVs each month. Participants were also given the opportunity to provide blood samples on filter paper (dried blood spot (DBS)) to be utilised in molecular and serological analyses described in this thesis. All participants or parents/guardians provided informed consent and ethical approval was obtained from the Ethics Review Committee on Medical Research involving Human Subjects, Myanmar Department of Medical Research (21/Ethics/2015) and the Alfred Hospital, Melbourne, Australia (95/15).

## Antibody determination

### Elution of serum from DBS

For DBS collected from the VHV cohort, serum was eluted as follows. Blood products were eluted from a single, 3mm circular punch taken from the centre of the DBS. Punches were incubated overnight at room temperature in 150µL Phosphate Buffered Saline (PBS) + 0.05% Tween20 and 0.05% Sodium Azide with gentle shaking, giving a final serum dilution of ~1:200. The eluted products were then aliquoted and stored at -80°C until use.

### Detection of Total IgG

Detection of IgG was carried out by enzyme-linked immunosorbent assay (ELISA) using a robotic liquid handling system (JANUS automated work station, Perkin Elmer) according to established, previously published protocols (2). Between each incubation and addition step, plates were washed three times with 1 x PBS + 0.05% Tween20 using an automated plate washer. Spectraplates were coated with 0.5μg/mL recombinant *P. falciparum* MSP-2 (3D7 whole ectodomain [19-249]), AMA1 (3D7 [25-546]), CSP (3D7 full length [1-398]) or *P. vivax* AMA1 (Palo Alto [1-471]). *Pf*AMA1, CSP and *Pv*AMA1 were expressed in the HEK293 mammalian cell system, and *Pf*MSP2 was expressed in *Escherichia coli* as described previously (3). Plates were incubated overnight at 4°C, blocked for 2 hours at room temperature with 1% casein in 1 x PBS, and then incubated with patient and control sera diluted 1:2000 in 0.1% casein PBS at room temperature for 2 hours. Goat anti-human Horse Radish Peroxidase (HRP) -conjugated antibody was added at concentration of 1:2000 diluted in 0.1% casein in 1 x PBS for one hour at room temperature. Tetramethylbenzidine (TMB) substrate was added to each well and covered for 15 minutes at room temperature, then stopped with 1M sulphuric acid, and read in a spectrophotometer at 450nm. Wells containing no test sera were used to deduct background reactivity from each sample, and a seropositivity cut-off point was set at an OD above the mean + 3SDs of a panel of Melbourne donors.

## Molecular detection of subclinical infections

Due to the large volume of DBS collected from participants in the VHV cohort, a two stage process for molecular determination of subclinical infections was used. Firstly, DNA was extracted in pools and used in assays to detect *Plasmodium* genus infections to eliminate the majority of *Plasmodium* spp. negative samples. Following this, DNA was extracted from individual samples and used in *Plasmodium* spp. reactions to detect *P. falciparum* and *P. vivax* infection as described below.

### qPCR detection of *Plasmodium* spp. infections

DNA was extracted from DBS pools consisting of a single 3mm punch from 10 individual participant samples using the FavorPrep 96-well genomic DNA extraction kit by vacuum manifold according to manufacturer’s instructions. DNA was eluted in 50µL elution buffer. Pooled DNA was then tested for generic *Plasmodium* spp. infection in simplex qPCR (summarised in Tables S1 and S2).

### qPCR detection of *P. falciparum* and *P. vivax* infections

DNA from samples included in a pool that were positive for *Plasmodium* spp. infection was then extracted from 2 x 3mm DBS punches and utilised in duplex qPCR for *P. falciparum* and *P. vivax* (Summarised in Tables S1 and S2) according to previously published methods (4). Standard curve for P. falciparum and P. vivax were made from a 10-fold serial dilution of the control plasmids ranging from 10^5^ copies/μL to 5 copies/μL in duplicate. Samples yielding threshold cycle values equal or higher than 40 were considered Plasmodium species negative. All assays were performed in 96-well plate format on a Stratagene x3005p lightcycler.

**References:**

1. Win Han O, Cutts JC, Agius PA, Kyaw Zayar A, Poe Poe A, Aung T, et al. Effectiveness of repellent delivered through village health volunteers on malaria incidence in villages in South-East Myanmar: a stepped-wedge cluster-randomised controlled trial protocol. BMC infectious diseases. 2018;18(1):663.

2. Charnaud SC, McGready R, Herten-Crabb A, Powell R, Guy A, Langer C, et al. Maternal-foetal transfer of Plasmodium falciparum and Plasmodium vivax antibodies in a low transmission setting. Scientific reports. 2016;6:20859.

3. Boyle MJ, Chan JA, Handayuni I, Reiling L, Feng G, Hilton A, et al. IgM in human immunity to Plasmodium falciparum malaria. Science advances. 2019;5(9):eaax4489.

4. Rosanas-Urgell A, Mueller D, Betuela I, Barnadas C, Iga J, Zimmerman PA, et al. Comparison of diagnostic methods for the detection and quantification of the four sympatric Plasmodium species in field samples from Papua New Guinea. Malar J. 2010;9:361.
