# Supplementary material for: Community-based molecular and serological surveillance of subclinical malaria in Myanmar
Source: BMC Med. 2021 May 28;19:121. doi: 10.1186/s12916-021-01993-8 (PMC8161608; doi:10.1186/s12916-021-01993-8)
Supplement: Supplementary file 2 — Additional file 2. Supplementary tables and figures. [file 12916_2021_1993_MOESM2_ESM.docx]

**Supplementary Tables and Figures**
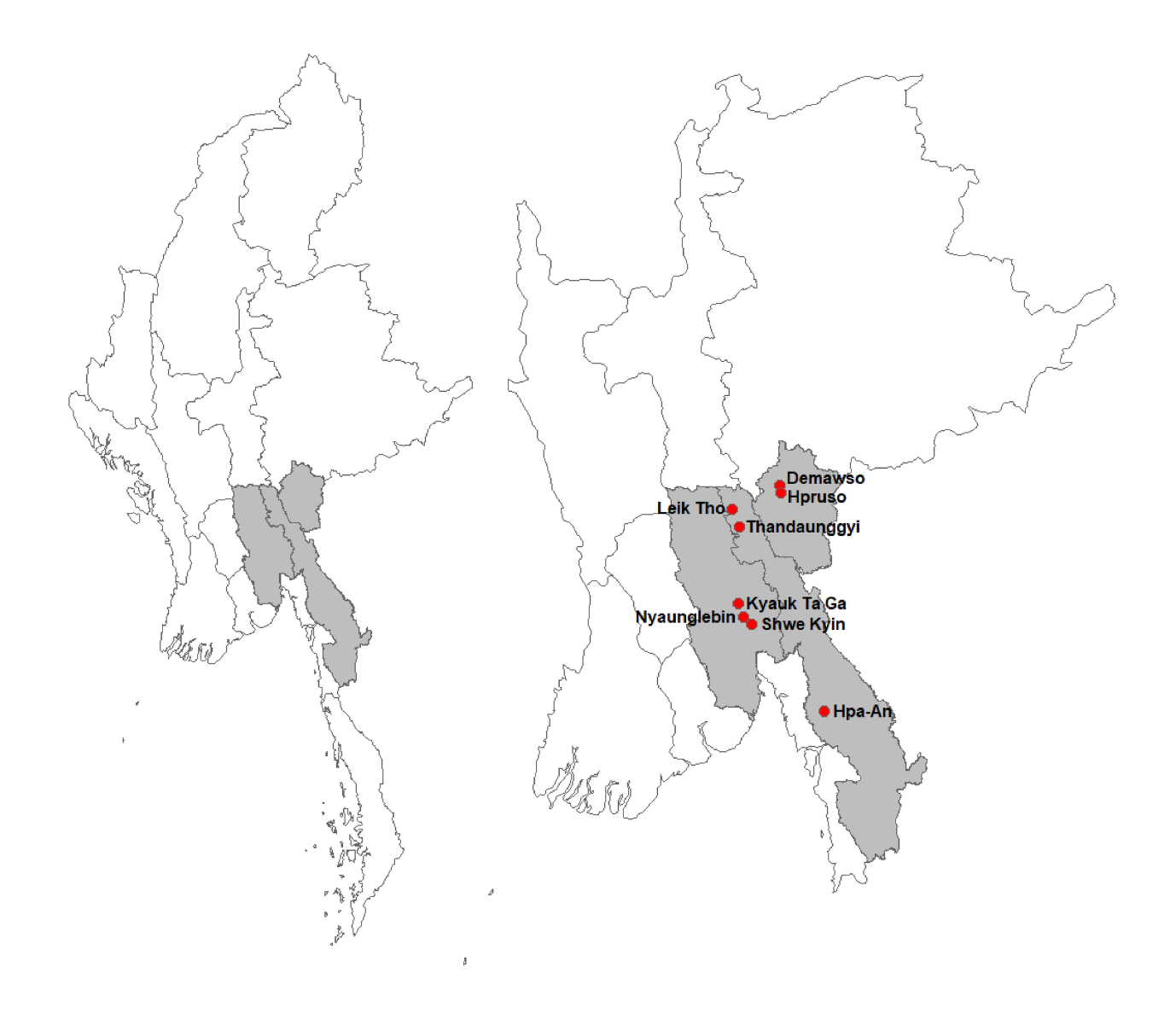


Figure S1 Location of participating townships in Southeast Myanmar. Map was generated using the tmap package in R with data from Natural Earth.

| **Cluster** | **Year/Month** | | | | | | | | | | | | | | |
| --- | --- | --- | --- | --- | --- | --- | --- | --- | --- | --- | --- | --- | --- | --- | --- |
|  | **2015 Apr** | **2015 May** | **2015**  **Jun** | **2015**  **Jul** | **2015**  **Aug** | **2015**  **Sep** | **2015**  **Oct** | **2015**  **Nov** | **2016**  **Apr** | **2016**  **May** | **2016**  **Jun** | **2016**  **Jul** | **2016**  **Aug** | **2016**  **Sep** | **2016**  **Oct** |
| VHV 1-8 | C | I | I | I | I | I | I | I | I | I | I | I | I | I | I |
| VHV 9-16 | C | C | I | I | I | I | I | I | I | I | I | I | I | I | I |
| VHV 17-24 | C | C | C | I | I | I | I | I | I | I | I | I | I | I | I |
| VHV 25-32 | C | C | C | C | I | I | I | I | I | I | I | I | I | I | I |
| VHV 33-40 | C | C | C | C | C | I | I | I | I | I | I | I | I | I | I |
| VHV 41-48 | C | C | C | C | C | C | I | I | I | I | I | I | I | I | I |
| VHV 49-56 | C | C | C | C | C | C | C | I | I | I | I | I | I | I | I |
| VHV 57-64 | C | C | C | C | C | C | C | C | I | I | I | I | I | I | I |
| VHV 65-72 | C | C | C | C | C | C | C | C | C | I | I | I | I | I | I |
| VHV 73-80 | C | C | C | C | C | C | C | C | C | C | I | I | I | I | I |
| VHV 81-88 | C | C | C | C | C | C | C | C | C | C | C | I | I | I | I |
| VHV 89-96 | C | C | C | C | C | C | C | C | C | C | C | C | I | I | I |
| VHV 97-104 | C | C | C | C | C | C | C | C | C | C | C | C | C | I | I |
| VHV 105-116 | C | C | C | C | C | C | C | C | C | C | C | C | C | C | I |

Figure S2 Stepped wedge cluster randomised controlled trial design for *Plasmodium* spp. infection and VHV topical insect repellent distribution.

Figure S3: Seroprevalence of anti-*P. falciparum* and *P. vivax* IgG by month over the study period by qPCR result. Seroprevalence (95% CI) of anti-*P. falciparum* and *P. vivax* IgG by month over the study period in qPCR *Plasmodium* spp. positive and negative individuals. High transmission season is shown in grey and months marked with an asterisk. Lines connecting data points are intended only to highlight patterns and not to suggest continuum between data points.

| **Table S1 qPCR primers and probes** | | | |
| --- | --- | --- | --- |
| **Species** | **Primer/probe** | **Sequence 5'-3'** | **Fluorescent label** |
| *Plasmodium* spp. | Forward | CTTCCTTAGATGTGGTAGCTATTTCTCA | FAM-BHQ |
|  | Reverse | ACATGGCTATGACGGGTAAC |  |
|  | Probe | FAMAATTAGAGTTCGATTC |  |
| *P. falciparum* | Forward | TATTGCTTTTGAGAGGTTTTGTTACTTTG | FAM-BHQ |
|  | Reverse | ACCTCTGACATCTGAATACGAATGC |  |
|  | Probe | ACGGGTAGTCATGATTGAGTT |  |
| *P. vivax* | Forward | GCTTTGTAATTGGAATGATGGGAAT | HEX-BHQ |
|  | Reverse | ATGCGCACAAAGTCGATACGAAG |  |
|  | Probe | AGCAACGCTTCTAGCTTA |  |

| **Table S2 qPCR reagents and cycling conditions** | | | | | | | |
| --- | --- | --- | --- | --- | --- | --- | --- |
| ***Plasmodium* spp.** | | | | ***P. falciparum* and *P. vivax*** | | |  |
| **µL/reaction** | | **Cycling parameters** | | **µL/reaction** | | **Cycling parameters** | |
| Taqman master mix | 10 | 95°C 2 minutes |  | Taqman master mix | 6.5 | 95°C 15 minutes |  |
| Forward primer | 0.8 |  |  | Forward primer | 0.2 |  |  |
| Reverse primer | 0.8 | 95°C 10 seconds | 40 cycles | Reverse primer | 0.2 | 95°C 15 seconds | 45 cycles |
| Probe | 0.2 | 60°C 50 seconds |  | Probe | 0.45 | 60°C 1 minute |  |
| Nuclease free water | 3.2 |  |  | Nuclease free water | 0.8 | 40°C 1 minute |  |

| Table S3 Number of participant repeated measurements | |
| --- | --- |
| No. of visits / measurements | **No. participants (% total measurements)** |
| Single | 8, 825 (81.28%) |
| Two | 1, 340 (12.34%) |
| Three | 382 (3.51%) |
| Four | 177 (1.63%) |
| Five | 69 (0.64%) |
| Six | 36 (0.31%) |
| Seven | 21 (0.19%) |
| Eight | 3 (0.03%) |
| Nine | 3 (0.03%) |
| Ten | 1 (0.01%) |
| TOTAL | **10, 857 (100%)** |

| **Table S4 Odds of qPCR detectable *P. falciparum* infection and anti-*Pf*AMA1 IgG seropositivity** | | | | | | | | | | | | |
| --- | --- | --- | --- | --- | --- | --- | --- | --- | --- | --- | --- | --- |
|  | **Contemporaneous Infection** | | | | | | **Infection at next presentation** | | | | | |
|  | **aOR** | | **(95%CI)** | | **p-value** | | **aOR** | **(95%CI)** | | | **p-value** | |
| *Pf*AMA1 IgG Seroprevalence | | | | | | |  | | | | | |
| Seronegative | REF | | | | | | REF | | | | | |
| Seropositive | 0.70 | | (0.52, 0.93) | | 0.01 | | 0.98 | | (0.53, 1.80) | | | 0.95 |
| Age (years) | 1.00 | | (0.99, 1.01) | | 0.57 | | 1.00 | | (0.98, 1.02) | | | 0.93 |
| Sex | | | | | | |  | | | | | |
| Female | REF | | | | | | REF | | | | | |
| Male | 0.93 | | (0.74, 1.18) | | 0.57 | | 0.67 | (0.38, 1.16) | | | 0.15 | |
| Region | | | | | | |  | | | | | |
| Hpa-An | REF | | | | | | REF | | | | | |
| Loikaw | 0.86 | | (0.58, 1.27) | | 0.45 | | 0.76 | | (0.36, 1.60) | | 0.47 | |
| Taungoo | 0.86 | | (0.60, 1.23) | | 0.40 | | 0.36 | | (0.17, 0.74) | | 0.01 | |
| Yangon | 0.97 | | (0.61, 1.55) | | 0.91 | | 0.34 | | (0.04, 2.71) | | 0.31 | |
| Residential status | | | | | | |  | | | | | |
| Village resident | REF | | | | | | REF | | | | | |
| Resident/forest dweller | 1.22 | | (0.92, 1.62) | | 0.17 | | 0.91 | (0.47, 1.75) | | 0.78 | | |
| Migrant | 1.26 | | (0.85, 1.86) | | 0.25 | | 2.15 | (0.72, 6.45) | | 0.17 | | |
| Time (month) | |  | | | | | - | | | | | |
| April 2015 | REF | | | | | |  |  |  |  |  |  |
| May | 0.64 | | (0.36, 1.13) | | | 0.12 |  |  |  |  |  |  |
| June | 1.40 | | (0.86, 2.29) | | | 0.18 |  |  |  |  |  |  |
| July | 0.91 | | (0.53, 1.57) | | | 0.74 |  |  |  |  |  |  |
| August | 0.07 | | (0.02, 0.30) | | | <0.001 |  |  |  |  |  |  |
| September | 0.25 | | (0.11, 0.59) | | | 0.001 |  |  |  |  |  |  |
| October | 0.03 | | (0.004, 0.22) | | | 0.001 |  |  |  |  |  |  |
| November | 0.10 | | (0.02, 0.41) | | | 0.002 |  |  |  |  |  |  |
| December | 1.59 | | (0.96, 2.62) | | | 0.07 |  |  |  |  |  |  |
| January 2016 | 0.46 | | (0.25, 0.84) | | | 0.01 |  |  |  |  |  |  |
| February | 1.08 | | (0.65, 1.80) | | | 0.76 |  |  |  |  |  |  |
| March | 0.56 | | (0.30, 1.06) | | | 0.08 |  |  |  |  |  |  |
| April | 0.30 | | (0.14, 0.63) | | | 0.001 |  |  |  |  |  |  |
| May | 0.54 | | (0.27, 1.08) | | | 0.08 |  |  |  |  |  |  |
| June | 0.93 | | (0.43, 2.00) | | | 0.85 |  |  |  |  |  |  |
| Repellent Distribution | | | | | | |  | | | | | |
| Not yet distributed | REF | | | | | | REF | | | | | |
| Distributed | 0.63 | | (0.47, 0.86) | | 0.003 | | 0.50 | (0.28, 0.89) | | 0.02 | | |
| Time since last measurement (days) | | | | - | | | 1.00 | | (0.99, 1.01) | | 0.11 | |
| aOR: adjusted odds ratio, 95%CI: 95% confidence interval, p-value: probability value | | | | | | | | | | | | |

| Table S5 Odds of qPCR detectable *P. falciparum* infection and anti-*Pf*AMA1 IgG level | | | | | | | | | | | |
| --- | --- | --- | --- | --- | --- | --- | --- | --- | --- | --- | --- |
|  | **Contemporaneous Infection** | | | | | | **Infection at next presentation** | | | | |
|  | **aOR** | **(95%CI)** | **p-value** | | | | **aOR** | **(95%CI)** | **p-value** | | |
| *Pf*AMA1 IgG Level (log_2_OD 450nm) | | 0.94 | | (0.84, 1.06) | | 0.34 | 0.91 | | (0.73, 1.10) | 0.33 | |
| Age (years) | | 1.00 | | (0.99, 1.01) | | 0.37 | 1.00 | | (0.98, 1.02) | 0.80 | |
| Sex | | | | | | |  | | | | |
| Female | | REF | | | | | REF | | | | |
| Male | | 0.96 | | (0.74, 1.24) | | 0.75 | 0.60 | | (0.33, 1.07) | 0.09 |  |
| Region | | | | | | |  | | | | |
| Hpa-An | | REF | | | | | REF | | | | |
| Loikaw | | 0.82 | | (0.54, 1.25) | | 0.36 | 0.75 | | (0.35, 1.60) | 0.45 | |
| Taungoo | | 0.79 | | (0.54, 1.16) | | 0.24 | 0.31 | | (0.15, 0.66) | 0.46 | |
| Yangon | | 0.79 | | (0.46, 1.36) | | 0.40 | 0.37 | | (0.05, 2.90) | 0.34 | |
| Residential status | | | | | | |  | | | | |
| Village resident | | REF | | | | | REF | | | | |
| Resident/forest dweller | | 1.21 | | (0.90, 1.65) | | 0.21 | 0.91 | | (0.46, 1.81) | 0.79 |  |
| Migrant | | 1.07 | | (0.69, 1.65) | | 0.76 | 2.30 | | (0.76, 6.94) | 0.14 |  |
| Time (month) | | |  | | | | - | | | | |
| April 2015 | | REF | | | | |  |  |  |  |  |
| May | | 0.61 | | (0.33, 1.12) | | 0.11 |  |  |  |  |  |
| June | | 1.32 | | (0.80, 2.20) | | 0.28 |  |  |  |  |  |
| July | | 0.91 | | (0.52, 1.61) | | 0.76 |  |  |  |  |  |
| August | | 0.08 | | (0.02, 0.32) | | <0.001 |  |  |  |  |  |
| September | | 0.24 | | (0.09, 0.64) | | 0.005 |  |  |  |  |  |
| October | | 0.03 | | (0.004, 0.25) | | 0.001 |  |  |  |  |  |
| November | | 0.11 | | (0.03, 0.47) | | 0.003 |  |  |  |  |  |
| December | | 1.76 | | (1.03, 3.02) | | 0.04 |  |  |  |  |  |
| January 2016 | | 0.54 | | (0.28, 1.04) | | 0.07 |  |  |  |  |  |
| February | | 1.10 | | (0.63, 1.90) | | 0.74 |  |  |  |  |  |
| March | | 0.64 | | (0.33, 1.27) | | 0.20 |  |  |  |  |  |
| April | | 0.10 | | (0.02, 0.43) | | 0.002 |  |  |  |  |  |
| May | | 0.61 | | (0.29, 1.29) | | 0.20 |  |  |  |  |  |
| June | | 1.10 | | (0.46, 2.25) | | 0.97 |  |  |  |  |  |
| Repellent Distribution | | | | | | |  | | | | |
| Not yet distributed | | REF | | | | | REF | | | | |
| Distributed | | 0.60 | | (0.43, 0.83) | | 0.002 | 0.46 | | (0.26, 0.84) | 0.01 |  |
| Time since last measurement (days) | | | | | - | | 1.00 | | (0.99, 1.01) | 0.09 | |
| aOR: adjusted odds ratio, 95%CI: 95% confidence interval | | | | | | | | | | | |

| Table S6 Odds of qPCR detectable *P. falciparum* infection and anti-*Pf*MSP2 IgG seropositivity | | | | | | | | | | |
| --- | --- | --- | --- | --- | --- | --- | --- | --- | --- | --- |
|  | **Contemporaneous Infection** | | | | | | **Infection at next presentation** | | | |
|  | **aOR** | | **(95%CI)** | | **p-value** | | **aOR** | **(95%CI)** | | **p-value** |
| *Pf*MSP2 IgG Seroprevalence | | | | | | |  | | | |
| Seronegative | REF | | | | | | REF | | | |
| Seropositive | 0.81 | | (0.61, 1.08) | | 0.15 | | 1.27 | (0.70, 2.29) | 0.43 | |
| Age (years) | 1.00 | | (0.99, 1.01) | | 0.26 | | 1.00 | (0.98, 1.02) | 0.82 | |
| Sex | | | | | | |  | | | |
| Female | REF | | | | | | REF | | | |
| Male | 0.93 | | (0.73, 1.17) | | 0.52 | | 0.66 | (0.38, 1.16) | | 0.15 |
| Region | | | | | | |  | | | |
| Hpa-An | REF | | | | | | REF | | | |
| Loikaw | 0.83 | | (0.56, 1.22) | | 0.34 | | 0.77 | (0.37, 1.61) | 0.49 | |
| Taungoo | 0.79 | | (0.55, 1.12) | | 0.18 | | 0.35 | (0.17, 0.72) | 0.005 | |
| Yangon | 0.96 | | (0.60, 1.53) | | 0.87 | | 0.36 | (0.05, 2.82) | 0.33 | |
| Residential status | | | | | | |  | | | |
| Village resident | REF | | | | | | REF | | | |
| Resident/forest dweller | 1.21 | | (0.91, 1.60) | | 0.19 | | 0.91 | (0.47, 1.75) | | 0.78 |
| Migrant | 1.25 | | (0.85, 1.84) | | 0.26 | | 2.12 | (0.71, 6.35) | | 0.18 |
| Time (month) | |  | | | | | - | | | |
| April 2015 | REF | | | | | |  |  |  |  |
| May | 0.63 | | (0.36, 1.11) | | | 0.11 |  |  |  |  |
| June | 1.18 | | (0.74, 1.91) | | | 0.49 |  |  |  |  |
| July | 0.82 | | (0.48, 1.41) | | | 0.48 |  |  |  |  |
| August | 0.07 | | (0.02, 0.32) | | | <0.001 |  |  |  |  |
| September | 0.27 | | (0.12, 0.62) | | | 0.002 |  |  |  |  |
| October | 0.03 | | (0.005, 0.24) | | | 0.001 |  |  |  |  |
| November | 0.11 | | (0.03, 0.46) | | | 0.003 |  |  |  |  |
| December | 1.61 | | (0.97, 2.65) | | | 0.06 |  |  |  |  |
| January 2016 | 0.48 | | (0.26, 0.87) | | | 0.02 |  |  |  |  |
| February | 1.25 | | (0.74, 2.10) | | | 0.41 |  |  |  |  |
| March | 0.65 | | (0.34, 1.23) | | | 0.19 |  |  |  |  |
| April | 0.35 | | (0.17, 0.74) | | | 0.006 |  |  |  |  |
| May | 0.60 | | (0.29, 1.20) | | | 0.15 |  |  |  |  |
| June | 0.99 | | (0.46, 2.14) | | | 0.98 |  |  |  |  |
| Repellent Distribution | | | | | | |  | | | |
| Not yet distributed | REF | | | | | | REF | | | |
| Distributed | 0.63 | | (0.47, 0.86) | | 0.003 | | 0.50 | (0.28, 0.89) | | 0.02 |
| Time since last measurement (days) | | | | - | | | 1.00 | (0.99, 1.01) | 0.09 | |
| aOR: adjusted odds ratio, 95%CI: 95% confidence interval | | | | | | | | | | |

| Table S7 Odds of qPCR detectable *P. falciparum* infection and anti-*Pf*MSP2 IgG level | | | | | | | | | | |
| --- | --- | --- | --- | --- | --- | --- | --- | --- | --- | --- |
|  | **Contemporaneous Infection** | | | | | | **Infection at next presentation** | | | |
|  | **aOR** | | **(95%CI)** | | **p-value** | | **aOR** | **(95%CI)** | | **p-value** |
| *Pf*MSP2 IgG Level (log_2_OD 450nm) | 0.88 | | (0.80, 0.96) | | 0.005 | | 0.92 | (0.77, 1.11) | 0.40 | |
| Age (years) | 0.99 | | (0.98, 1.00) | | 0.21 | | 0.99 | (0.98, 1.02) | 0.92 | |
| Sex | | | | | | |  | | | |
| Female | REF | | | | | | REF | | | |
| Male | 0.93 | | (0.73, 1.18) | | 0.54 | | 0.69 | (0.39, 1.20) | | 0.19 |
| Region | | | | | | |  | | | |
| Hpa-An | REF | | | | | | REF | | | |
| Loikaw | 0.81 | | (0.55, 1.20) | | 0.29 | | 0.74 | (0.35, 1.56) | 0.43 | |
| Taungoo | 0.80 | | (0.56, 1.13) | | 0.21 | | 0.38 | (0.18, 0.80) | 0.01 | |
| Yangon | 0.90 | | (0.56, 1.45) | | 0.68 | | 0.39 | (0.05, 3.04) | 0.37 | |
| Residential status | | | | | | |  | | | |
| Village resident | REF | | | | | | REF | | | |
| Resident/forest dweller | 1.25 | | (0.94, 1.66) | | 0.13 | | 0.89 | (0.46, 1.73) | | 0.73 |
| Migrant | 1.29 | | (0.87, 1.91) | | 0.21 | | 2.12 | (0.71, 6.37) | | 0.18 |
| Time (month) | |  | | | | | - | | | |
| April 2015 | REF | | | | | |  |  |  |  |
| May | 0.62 | | (0.35, 1.12) | | | 0.11 |  |  |  |  |
| June | 1.18 | | (0.72, 1.91) | | | 0.51 |  |  |  |  |
| July | 0.76 | | (0.44, 1.34) | | | 0.35 |  |  |  |  |
| August | 0.08 | | (0.02, 0.32) | | | <0.001 |  |  |  |  |
| September | 0.28 | | (0.12, 0.64) | | | 0.003 |  |  |  |  |
| October | 0.03 | | (0.004, 0.24) | | | 0.001 |  |  |  |  |
| November | 0.12 | | (0.03, 0.51) | | | 0.004 |  |  |  |  |
| December | 1.62 | | (0.97, 2.70) | | | 0.07 |  |  |  |  |
| January 2016 | 0.52 | | (0.28, 0.94) | | | 0.03 |  |  |  |  |
| February | 1.35 | | (0.80, 2.30) | | | 0.26 |  |  |  |  |
| March | 0.73 | | (0.38, 1.39) | | | 0.34 |  |  |  |  |
| April | 0.33 | | (0.15, 0.74) | | | 0.007 |  |  |  |  |
| May | 0.67 | | (0.33, 1.35) | | | 0.26 |  |  |  |  |
| June | 1.05 | | (0.49, 2.29) | | | 0.89 |  |  |  |  |
| Repellent Distribution | | | | | | |  | | | |
| Not yet distributed | REF | | | | | | REF | | | |
| Distributed | 0.64 | | (0.47, 0.87) | | 0.004 | | 0.52 | (0.29, 0.93) | | 0.03 |
| Time since last measurement (days) | | | | - | | | 1.00 | (0.99, 1.01) | 0.12 | |
| aOR: adjusted odds ratio, 95%CI: 95% confidence interval | | | | | | | | | | |

| Table S8 Odds of qPCR detectable *P. falciparum* infection and anti-*Pf*CSP IgG seropositivity | | | | | | | | | | |
| --- | --- | --- | --- | --- | --- | --- | --- | --- | --- | --- |
|  | **Contemporaneous Infection** | | | | | | **Infection at next presentation** | | | |
|  | **aOR** | | **(95%CI)** | | **p-value** | | **aOR** | **(95%CI)** | | **p-value** |
| *Pf*CSP IgG Seroprevalence | | | | | | |  | | | |
| Seronegative | REF | | | | | | REF | | | |
| Seropositive | 0.51 | | (0.35, 0.76) | | 0.001 | | 0.29 | (0.07, 1.21) | 0.09 | |
| Age (years) | 0.99 | | (0.98, 1.01) | | 0.23 | | 1.00 | (0.98, 1.02) | 0.98 | |
| Sex | | | | | | |  | | | |
| Female | REF | | | | | | REF | | | |
| Male | 0.92 | | (0.73, 1.17) | | 0.50 | | 0.67 | (0.38, 1.16) | | 0.15 |
| Region | | | | | | |  | | | |
| Hpa-An | REF | | | | | | REF | | | |
| Loikaw | 0.84 | | (0.57, 1.23) | | 0.37 | | 0.77 | (0.37, 1.62) | 0.49 | |
| Taungoo | 0.80 | | (0.56, 1.14) | | 0.21 | | 0.37 | (0.18, 0.76) | 0.01 | |
| Yangon | 0.98 | | (0.62, 1.57) | | 0.95 | | 0.35 | (0.04, 2.74) | 0.32 | |
| Residential status | | | | | | |  | | | |
| Village resident | REF | | | | | | REF | | | |
| Resident/forest dweller | 1.20 | | (0.91, 1.59) | | 0.20 | | 0.91 | (0.47, 1.75) | | 0.77 |
| Migrant | 1.25 | | (0.85, 1.84) | | 0.27 | | 2.18 | (0.73, 6.55) | | 0.16 |
| Time (month) | |  | | | | | - | | | |
| April 2015 | REF | | | | | |  |  |  |  |
| May | 0.62 | | (0.35, 1.09) | | | 0.10 |  |  |  |  |
| June | 1.21 | | (0.75, 1.94) | | | 0.44 |  |  |  |  |
| July | 0.81 | | (0.47, 1.39) | | | 0.45 |  |  |  |  |
| August | 0.07 | | (0.02, 0.30) | | | <0.001 |  |  |  |  |
| September | 0.26 | | (0.11, 0.60) | | | 0.002 |  |  |  |  |
| October | 0.03 | | (0.004, 0.23) | | | 0.001 |  |  |  |  |
| November | 0.10 | | (0.02, 0.43) | | | 0.002 |  |  |  |  |
| December | 1.56 | | (0.95, 2.57) | | | 0.08 |  |  |  |  |
| January 2016 | 0.51 | | (0.28, 0.92) | | | 0.03 |  |  |  |  |
| February | 1.19 | | (0.71, 1.97) | | | 0.51 |  |  |  |  |
| March | 0.65 | | (0.35, 1.22) | | | 0.18 |  |  |  |  |
| April | 0.42 | | (0.20, 0.88) | | | 0.02 |  |  |  |  |
| May | 0.57 | | (0.28, 1.13) | | | 0.11 |  |  |  |  |
| June | 0.94 | | (0.44, 2.03) | | | 0.88 |  |  |  |  |
| Repellent Distribution | | | | | | |  | | | |
| Not yet distributed | REF | | | | | | REF | | | |
| Distributed | 0.64 | | (0.47, 0.87) | | 0.004 | | 0.54 | (0.30, 0.95) | | 0.03 |
| Time since last measurement (days) | | | | - | | | 1.00 | (0.99, 1.01) | 0.16 | |
| aOR: adjusted odds ratio, 95%CI: 95% confidence interval | | | | | | | | | | |

| Table S9 Odds of qPCR detectable *P. falciparum* infection and anti-*Pf*CSP IgG level | | | | | | | | | | |
| --- | --- | --- | --- | --- | --- | --- | --- | --- | --- | --- |
|  | **Contemporaneous Infection** | | | | | | **Infection at next presentation** | | | |
|  | **aOR** | | **(95%CI)** | | **p-value** | | **aOR** | **(95%CI)** | | **p-value** |
| *Pf*CSP IgG Level (log_2_OD 450nm) | 0.90 | | (0.83, 0.98) | | 0.02 | | 1.02 | (0.85, 1.24) | 0.81 | |
| Age (years) | 0.99 | | (0.98, 1.00) | | 0.12 | | 1.00 | (0.98, 1.02) | 0.66 | |
| Sex | | | | | | |  | | | |
| Female | REF | | | | | | REF | | | |
| Male | 0.91 | | (0.70, 1.19) | | 0.49 | | 0.63 | (0.34, 1.16) | | 0.14 |
| Region | | | | | | |  | | | |
| Hpa-An | REF | | | | | | REF | | | |
| Loikaw | 0.75 | | (0.49, 1.17) | | 0.21 | | 0.72 | (0.33, 1.59) | 0.42 | |
| Taungoo | 0.77 | | (0.52, 1.14) | | 0.19 | | 0.30 | (0.14, 0.67) | 0.003 | |
| Yangon | 0.70 | | (0.40, 1.25) | | 0.23 | | 0.34 | (0.04, 2.73) | 0.31 | |
| Residential status | | | | | | |  | | | |
| Village resident | REF | | | | | | REF | | | |
| Resident/forest dweller | 1.26 | | (0.92, 1.73) | | 0.15 | | 0.97 | (0.47, 2.00) | | 0.94 |
| Migrant | 0.96 | | (0.59, 1.54) | | 0.85 | | 2.62 | (0.86, 8.01) | | 0.09 |
| Time (month) | |  | | | | | - | | | |
| April 2015 | REF | | | | | |  |  |  |  |
| May | 0.62 | | (0.35, 1.10) | | | 0.10 |  |  |  |  |
| June | 1.21 | | (0.74, 1.99) | | | 0.45 |  |  |  |  |
| July | 0.71 | | (0.40, 1.26) | | | 0.25 |  |  |  |  |
| August | 0.07 | | (0.02, 0.29) | | | <0.001 |  |  |  |  |
| September | 0.26 | | (0.11, 0.61) | | | 0.002 |  |  |  |  |
| October | 0.03 | | (0.003, 0.22) | | | 0.001 |  |  |  |  |
| November | 0.11 | | (0.03, 0.47) | | | 0.003 |  |  |  |  |
| December | 1.28 | | (0.67, 2.44) | | | 0.46 |  |  |  |  |
| January 2016 | 0.46 | | (0.24, 0.89) | | | 0.02 |  |  |  |  |
| February | 0.96 | | (0.55, 1.66) | | | 0.87 |  |  |  |  |
| March | 0.54 | | (0.27, 1.05) | | | 0.07 |  |  |  |  |
| April | 0.29 | | (0.13, 0.65) | | | 0.003 |  |  |  |  |
| May | 0.48 | | (0.22, 1.06) | | | 0.07 |  |  |  |  |
| June | 0.75 | | (0.30, 1.88) | | | 0.54 |  |  |  |  |
| Repellent Distribution | | | | | | |  | | | |
| Not yet distributed | REF | | | | | | REF | | | |
| Distributed | 0.63 | | (0.44, 0.90) | | 0.01 | | 0.60 | (0.32, 1.11) | | 0.10 |
| Time since last measurement (days) | | | | - | | | 1.00 | (0.99, 1.01) | 0.12 | |
| aOR: adjusted odds ratio, 95%CI: 95% confidence interval, p-value: probability value | | | | | | | | | | |

| Table S10 Odds of qPCR detectable *P. vivax* infection and anti-*Pv*AMA1 IgG seropositivity | | | | |
| --- | --- | --- | --- | --- |
|  | **Contemporaneous Infection** | | | |
|  | **aOR** | | **(95%CI)** | **p-value** |
| *Pv*AMA1 IgG Seroprevalence | | | | |
| Seronegative | REF | | | |
| Seropositive | 1.02 | | (0.73, 1.43) | 0.89 |
| Age (years) | 1.00 | | (0.99, 1.01) | 0.59 |
| Sex | | | | |
| Female | REF | | | |
| Male | 1.03 | | (0.78, 1.36) | 0.81 |
| Region | | | | |
| Hpa-An | REF | | | |
| Loikaw | 0.82 | | (0.50, 1.34) | 0.43 |
| Taungoo | 0.93 | | (0.60, 1.46) | 0.75 |
| Yangon | 1.31 | | (0.75, 2.29) | 0.35 |
| Residential status | | | | |
| Village resident | REF | | | |
| Resident/forest dweller | 1.04 | | (0.75, 1.45) | 0.80 |
| Migrant | 0.91 | | (0.57, 1.45) | 0.70 |
| Time (month) | |  | | |
| April 2015 | REF | | | |
| May | 0.47 | | (0.23, 0.96) | 0.04 |
| June | 1.74 | | (1.05, 2.88) | 0.03 |
| July | 1.76 | | (1.05, 2.97) | 0.03 |
| August | 0.14 | | (0.04, 0.46) | 0.001 |
| September | 0.91 | | (0.48, 1.71) | 0.78 |
| October | 0.35 | | (0.16, 0.77) | 0.01 |
| November | 0.11 | | (0.03, 0.48) | 0.003 |
| December | 0.26 | | (0.11, 0.63) | 0.003 |
| January 2016 | 0.02 | | (0.003, 0.17) | <0.001 |
| February | 0.16 | | (0.07, 0.40) | <0.001 |
| March | 0.29 | | (0.13, 0.65) | 0.003 |
| April | 0.03 | | (0.003, 0.20) | <0.001 |
| May | 0.53 | | (0.25, 1.09) | 0.08 |
| June | 0.55 | | (0.22, 1.36) | 0.20 |
| Repellent Distribution | | | | |
| Not yet distributed | REF | | | |
| Distributed | 1.06 | | (0.72, 1.56) | 0.76 |
| aOR: adjusted odds ratio, 95%CI: 95% confidence interval | | | | |

| Table S11 Odds of qPCR detectable *P. vivax* infection and anti-*Pv*AMA1 IgG level | | | | |
| --- | --- | --- | --- | --- |
|  | **Contemporaneous Infection** | | | |
|  | **aOR** | | **(95%CI)** | **p-value** |
| *Pv*AMA1 IgG Level (log_2_OD 450nm) | 0.98 | | (0.86, 1.12) | 0.75 |
| Age (years) | 1.00 | | (0.99, 1.01) | 0.58 |
| Sex | | | | |
| Female | REF | | | |
| Male | 1.03 | | (0.78, 1.36) | 0.85 |
| Region | | | | |
| Hpa-An | REF | | | |
| Loikaw | 0.83 | | (0.50, 1.35) | 0.45 |
| Taungoo | 0.95 | | (0.61, 1.49) | 0.83 |
| Yangon | 1.27 | | (0.72, 2.24) | 0.40 |
| Residential status | | | | |
| Village resident | REF | | | |
| Resident/forest dweller | 1.05 | | (0.75, 1.46) | 0.78 |
| Migrant | 0.92 | | (0.57, 1.46) | 0.72 |
| Time (month) | |  | | |
| April 2015 | REF | | | |
| May | 0.47 | | (0.23, 0.96) | 0.04 |
| June | 1.70 | | (1.02, 2.82) | 0.04 |
| July | 1.70 | | (1.00, 2.90) | 0.05 |
| August | 0.13 | | (0.04, 0.45) | 0.001 |
| September | 0.89 | | (0.47, 1.68) | 0.72 |
| October | 0.34 | | (0.15, 0.75) | 0.01 |
| November | 0.11 | | (0.02, 0.47) | 0.003 |
| December | 0.29 | | (0.12, 0.70) | 0.01 |
| January 2016 | 0.02 | | (0.004, 0.21) | 0.001 |
| February | 0.16 | | (0.07, 0.40) | <0.001 |
| March | 0.30 | | (0.13, 0.67) | 0.003 |
| April | 0.03 | | (0.004, 0.21) | 0.001 |
| May | 0.51 | | (0.24, 1.07) | 0.07 |
| June | 0.54 | | (0.22, 1.34) | 0.19 |
| Repellent Distribution | | | | |
| Not yet distributed | REF | | | |
| Distributed | 1.06 | | (0.72, 1.56) | 0.75 |
| aOR: adjusted odds ratio, 95%CI: 95% confidence interval | | | | |
